# Socio-demographic characteristics, lifestyles, social support quality and mental health in college students: a cross-sectional study

**DOI:** 10.1186/s12889-022-14002-1

**Published:** 2022-08-20

**Authors:** Chao Wang, Shijiao Yan, Heng Jiang, Yingying Guo, Yong Gan, Chuanzhu Lv, Zuxun Lu

**Affiliations:** 1 Department of Social Medicine and Health Management, School of Public Health, Wuhan University, No. 115 Donghu Road, Wuchang District, Wuhan, 430071 China; 2 Department of Emergency Medicine, Hunan Provincial Institute of Emergency Medicine, Hunan Provincial Key Laboratory of Emergency and Critical Care Metabolomics, Hunan Provincial People’s Hospital/The First Affiliated Hospital, Hunan Normal University, Changsha, 410081 Hunan China; 3 School of Public Health, Hainan Medical University, Haikou, 571199 Hainan China; 4 Centre for Alcohol Policy Research, School of Psychology and Public Health, La Trobe University, Melbourne, VIC 3086 Australia; 5 Centre for Health Equity, Melbourne School of Population and Global Health, The University of Melbourne, Melbourne, VIC 3010 Australia; 6 Department of Children’s Healthcare, Tongji Hospital, Tongji Medical College, Huazhong University of Science and Technology, Wuhan, 430030 Hubei China; 7 Department of Social Medicine and Health Management, School of Public Health, Tongji Medical College, Huazhong University of Science and Technology, No. 13 Hangkong Road, Qiaokou District, Wuhan, 430030 Hubei China; 8 Department of Emergency, the Second Affiliated Hospital of Hainan Medical University, Haikou, 570311 Hainan China; 9 Emergency and Trauma College, Hainan Medical University, Haikou, 570311 Hainan China

**Keywords:** Mental health problems, Social support, College students, Influencing factors, China

## Abstract

**Background:**

Mental health problems are important public health issues among college students and are associated with various social factors. However, these influencing factors were scarcely summarized in Chinese college students comprehensively. This study aims to assess the associations between socio-demographic characteristics, lifestyles, social support quality (SSQ) and mental health among Chinese college students .

**Methods:**

A cross-sectional study was conducted in Wuhan, China, from October 2017 to February 2018. College students from 18 colleges or universities were randomly recruited using multi-stage cluster sampling method. The Multidimensional Scale of Perceived Social Support scale and 12-items General Health Questionnaire were used to estimate students’ SSQ and mental health statuses, respectively. Logistic regression analysis was used to evaluate the associations between socio-demographic characteristics, lifestyles, SSQ and mental health problems.

**Results:**

A total of 10,676 college students were included. Among them, 21.4% were identified as having possible mental health problems. Students being a female, aged 18–22 years old, whose mother held college degrees and above, and drinking alcohol were more likely to have mental health problems (*P* < 0.05). Contrarily, having general or higher household economic levels, work-rest regularly, and sleeping ≥ 7 h were preventive factors (*P* < 0.05). Especially, a decreasing trend in the risk of having mental health problems with the improvement of SSQ was identified.

**Conclusion:**

Besides socio-demographic and lifestyle factors, social support is a critical factor for mental health among college students. Improving SSQ, especially which from the family, could be an effective method to prevent mental health problems among college students.

**Supplementary Information:**

The online version contains supplementary material available at 10.1186/s12889-022-14002-1.

## Background

Mental health problems are significant and growing public health issues, their high prevalence and heavy burdens have aroused people’s attention. A systematic review based on 174 studies across 63 countries suggested the 12-month common mental disorder prevalence was 17.6% and the lifetime prevalence was 29.2% [1]. Unfortunately, the effect of mental health problems can be long-lasting or recurrent.

Student’s mental health is an important topic throughout the education system, which not only affects students’ academic performance, but is a significant predictor of personal development [2]. Previous studies on students’ mental health problems mostly focused on the primary and secondary school years [3, 4]. However, it is also a prominent health problem among college students. Most of college students are just entering adulthood period, it is a crucial time for personal identity development and psychology transition. During this period, they are generally sensitive to the shift of surroundings, such as changes of living and learning environments [5, 6]. On the other hand, entering college/university is generally followed by considerable academic pressure and more adult-like responsibilities, but they may lack cognitive maturity or foundational skills required for adulthood [7]. A mental health survey performed by WHO in 21 countries showed that 20.3% of college students had suffered from mental health problems, but only 16.4% of them received appropriate healthcare [8]. China has the largest number of college students in the world and mental health problems are prominent health challenges for them, 16–30% college students have suffered from depression, anxiety, or other mental health problems [9]. Research documented that female college students usually showed a lower adjustment to college/university life and higher levels of worry and physiological sensitivity than males. Some studies also suggested the average prevalence of depression in college students was 30.39% [10], and the prevalence of anxiety was around 40% for male and 45% for female students [11]. While, the prevalence of depression [11], substance use [12] and physical violence were higher in males than in females.

Mental health is affected by complex reasons, such as socio-demographic characteristics [13], lifestyles [14], occupational status [15], as well as self-rated health [16] and social networks [17, 18]. Social support quality (SSQ) is considered as another critical influencing factor for mental health status [19], and dissatisfaction with insufficient or poor-quality social supports is closely associated with mental health problems [20]. Social support refers to the help provided by individuals who comprise the social network of a person who occupies the position of ego in this network [20], its quality may vary due to the source, intensity and frequency of social contacts, and family and friends seem to be the main sources of high-quality social support for students.

Although there were some attempts to estimate the prevalence and influencing factors of mental health among college students and provided general knowledge of their relationship, the sample sizes of these studies were relatively small [21, 22], or only focused on a specific dimension rather than comprehensive studies. In addition, previous studies have no clear result in comparing the effect magnitude of social supports from different sources for mental health. Therefore, we have two hypotheses: firstly, mental health of college students has significant relationships with socio-demographic characteristics, lifestyles and SSQ; secondly, mental health of college students shows an improving trend with the increase of SSQ, regardless of its source. To confirm the hypotheses, we conducted a large-scale epidemiological study among Chinese college students with two objectives. Firstly, we aimed to analyze the influencing factors of mental health problems among Chinese college students; secondly, we sought to evaluate the associations between college students’ mental health -statuses and SSQ from different sources.

## Methods

### Participants

We conducted a large population-based, cross-sectional study among 18 colleges/universities in Wuhan, China, from October 2017 to February 2018. In China, high school students who took the National College Entrance Exam could choose according to their grades and would be enrolled by different levels of colleges/universities. In general, the level of university is higher and its discipline settings are more complete than those of the college. Colleges/universities could be classified as comprehensive or specialized according to the discipline settings. All universities and colleges in China generally contain both male students and female students.

A multi-stage cluster random sampling method was applied in this survey. Firstly, according to subject settings, we categorized the 18 colleges/universities into seven groups: five comprehensive universities, seven universities of science and technology, two universities of finance and economics, and one university of teacher-training, agronomy, nationalities as well as sports. Secondly, we randomly selected, in the proportion of students sizes, several classes from each grade (from undergraduate to doctoral degree) in every college/university. Then, all students in selected classes were encouraged to participate in this survey with the voluntary principle, but college students who refused to sign or provide the informed consent were not included, and ensured no less than 500 questionnaires were received from each college/university. All participating students were asked to fulfill an online questionnaire on their computers or cellphones, which might take 5–15 mins to complete. The questionnaire was used to collect students’ information including socio-demographic characteristics, lifestyles or behaviors, perceived social support, and mental health statuses. Ultimately, a total of 11,750 college students participated in this survey and 11,093 questionnaires were collected on a computer terminal, with a response rate of 94.41%. After excluding those completed in less than five minutes, 10,676 qualified questionnaires were included in final statistical analyses, yielding a 96.24% qualification rate.

This study was approved by the ethics committee of Tongji Medical College institutional review board, Huazhong University of Science and Technology, Wuhan, China. All participants signed informed consent before filling out the questionnaire.

## Instruments

### Social support

Multidimensional Scale of Perceived Social Support (MSPSS) [23] consists of 12 items with response options scoring from 1 (very strongly disagree) to 7 (very strongly agree). It estimates SSQ from three sources: family (item 3, 4, 8, and 11), friends (item 6, 7, 9, and 12) and significant others (item 1, 2, 5, and 10) [24]. Scores of all items are added up and then divided by 12. The mean scores ranging from 1 to 2.99, 3 to 5 and 5.01 to 7 are classified as low, moderate, and high perceived support levels, respectively [23]. MSPSS has a sound factorial validity (with Cronbach’s alpha coefficients of 0.953), and internal consistencies for the full scale and subscales are both satisfactory [25]. The Chinese version has been suggested as a reliable tool for assessing SSQ [26].

### Mental health status

The Chinese version of 12-items General Health Questionnaire (GHQ-12) [27] has been used to measure mental health status in this study. The GHQ-12 has been widely used to screen individuals for minor mental disorders in the general population [28], it includes 12 items corresponding to three dimensions: anxiety/depression (item 1, 2, 7, and 10), social dysfunction (item 3, 4, 5, 6, 8, and 9) and deficiency of confidence (item 11 and 12) [29]. There are four answers ranging from “better/healthier than normal” to “much worse/more than usual”. The GHQ scoring method (the four options were scored by 0–0-1–1, respectively) has been adopted in our study. Higher score corresponds to worse mental health status. A total score of 4 or more was classified as having notable mental health problems [30]. GHQ-12 had satisfactory reliability (with Cronbach’s alpha coefficients of 0.886) and extensive sensitivity, its effectiveness for determining the prevalence of psychological disturbances has also been previously validated [31].

### Socio-demographic characteristics and lifestyles

The questionnaire includes the following socio-demographic variables: age, gender, ethnicity, religious belief, place of residence, from a single parent family or not, from a single child family or not, paternal/maternal education level, and household economic status. Household economic status was assessed by asking the question of “what do you think of your household economic condition?” with optional responses of “very affluent”, “more affluent”, “the general”, “less affluent”, or “non-affluent”. According to responses, household economic status was categorized as good, general, and poor.

Lifestyle variables refer to physical exercise, regular work-rest or not, sleep duration, smoking and alcohol drinking in this study. Physical exercise was judged from the question of “do you have chronic aerobic exercise (e.g. setting-up exercise, jogging, walking) for 30 min and longer three times a week?”, and the responses include “never/seldom”, “sometimes”, and “usually/always”. Regular work-rest was estimated by the question of “do you have a regular daily routine?”, and the options were also classified into three categories: “never/seldom”, “sometimes”, and “usually/always”. Sleep duration was divided into “ < 7 h”, “7–8.9 h”, and “ ≥ 9 h” based participants’ answers to “In recent three months, you sleep for XX hours, XX minutes every day on average.” Smoking and alcohol drinking were dichotomized as “yes” and “no” according to participants’ responses. Of them, smoking was defined as smoking at least one cigarette per week in the last 3 months, and alcohol drinking was defined as drinking alcohol at least once per month.

### Statistical analysis

Data analyses were performed using the SPSS software (Version 22 for Windows, SPSS Inc, Chicago, IL, U.S.A.). Descriptive analyses included means (standard deviations [SDs]) for continuous variables and frequencies (percentages) for categorical data. We analyzed respondents' demographic characteristics, and compared the differences of SSQ and mental health statuses among various demographics by *χ*^*2*^ tests. Potential influencing factors of mental health problems were identified via multivariate logistic regression analyses. Potential confounders included age, gender, ethnicity, religious belief, residence area, from a single parent family or not, from a single child family or not, parental education level, household economic status, physical exercise, work-rest routine, sleep duration, alcohol drinking, and smoking. In addition, we described the correlation between MSPSS and GHQ-12 by matrix analysis and estimated relationships between SSQ and mental health problems under different adjustments using trend analysis. Besides, we also explored gender differences in above analyses. Significance level was accepted as *P* < 0.05 (two-tailed) for all tests.

## Results

### Influencing factors of mental health

Referring to Table 1, among the included 10,676 college students (with a mean age of 19.66 [SD = 2.22]), 56.7% were female, and 2284 (21.4%) students had elevated scores on mental health questionnaires, suggesting possible mental health problems. Females, ethnic minority students, and students aged 18–21 years old, having religious beliefs, living in rural areas, from single parent families or non-single child families, whose parents’ education levels were primary or below, and from families with poorer economic status were more likely to have mental health problems (*P* < 0.05). For males, college students who are ethnic minority, from non-single child families, and with poorer household economic statuses had higher risk of having mental health problems (*P* < 0.05); For females, college students who are 18–25 years old, having religious beliefs, from urban areas, from single parent families, and whose mother having elementary school and below or college and above education levels, and those having poorer household economic status were vulnerable to mental health problems (*P* < 0.05).

**Table 1:** Demographic characteristic of participants by mental health problems

**Demographics**		**Total**				**Males**				**Females**			
		**Sample size (%)**	**Normal**	**Problematic**	***P***	**Sample size (%)**	**Normal**	**Problematic**	***P***	**Sample size (%)**	**Normal**	**Problematic**	***P***
**Age (years old)**^a^	< 18	680 (6.4)	81.8	18.2	< 0.001	271 (5.9)	81.5	18.5	0.593	409 (6.8)	81.9	18.1	0.003
	18–21	8136 (76.3)	77.9	22.1		3650 (78.9)	80.5	19.5		4486 (74.1)	75.8	24.2	
	22–25	1640 (15.4)	80.1	19.9		612 (13.2)	81.4	18.6		1028 (17.0)	79.3	20.7	
	≥ 26	203 (1.9)	84.2	15.8		86 (1.9)	86.0	14.0		117 (1.9)	82.9	17.1	
**Ethnicity**	Han	9476 (88.8)	79.1	20.9	0.985	4205 (90.9)	81.3	18.7	0.006	5217 (87.1)	77.3	22.7	0.054
	Minority	1200 (11.2)	74.8	25.3		420 (9.1)	75.7	24.3		780 (12.9)	74.2	25.8	
**Religious belief**	No	10,097 (94.6)	78.9	21.1	0.001	4345 (93.9)	81.0	19.0	0.151	5752 (95.1)	77.3	22.7	0.003
	Yes	579 (5.4)	73.6	26.4		280 (6.1)	77.5	22.5		299 (4.9)	69.9	30.1	
**Residence area**	Rural	4978 (46.6)	77.7	22.3	< 0.001	2326 (50.3)	81.1	18.9	0.546	3372 (55.7)	78.2	21.8	0.008
	Urban	5698 (53.4)	79.4	20.6		2299 (49.7)	80.4	19.6		2679 (44.3)	75.3	24.7	
**From a single parent family or not**	Yes	817 (7.7)	73.2	26.8	< 0.001	317 (6.9)	77.3	22.7	0.102	500 (8.3)	70.6	29.4	< 0.001
	No	9859 (92.3)	79.1	20.9		4308 (93.1)	81.0	19.0		5551 (91.7)	77.5	22.5	
**From a single child family or not**	Yes	4931 (46.2)	79.9	20.1	< 0.001	2397 (51.8)	82.5	17.5	0.002	2534 (41.9)	77.5	22.5	0.380
	No	5745 (53.8)	77.5	22.5		2228 (48.2)	78.9	21.1		3517 (58.1)	76.5	23.5	
**Paternal education level**	Elementary school and below	1558 (14.6)	76.4	23.6	< 0.001	711 (15.4)	78.9	21.1	0.251	847 (14.0)	74.4	25.6	0.127
	Junior high school	3872 (36.3)	78.7	21.3		1745 (37.7)	80.6	19.4		2127 (35.2)	77.1	22.9	
	High/Secondary school	2739 (25.7)	80.2	19.8		1152 (24.9)	82.6	17.4		1587 (26.2)	78.6	21.4	
	College and above	2507 (23.5)	78.1	21.9		1017 (22.0)	80.3	19.7		1490 (24.6)	76.5	23.5	
**Maternal education level**	Elementary school and below	2742 (25.7)	76.6	23.4	< 0.001	1275 (27.6)	78.4	21.6	0.065	1467 (24.2)	75.1	24.9	0.003
	Junior high school	3711 (34.8)	79.9	20.1		1646 (35.6)	82.3	17.7		2065 (34.1)	78.0	22.0	
	High/Secondary school	2395 (22.4)	80.0	20.0		983 (21.3)	80.8	19.2		1412 (23.3)	79.5	20.5	
	College and above	1828 (17.1)	77.1	22.9		721 (15.6)	81.4	18.6		1107 (18.3)	74.3	25.7	
**Household economic status**	Poor	3358 (31.5)	73.8	26.2	< 0.001	1624 (35.1)	77.0	23.0	< 0.001	1734 (28.7)	70.9	29.1	< 0.001
	General	6373 (59.7)	80.7	19.3		2627 (56.8)	82.7	17.3		3746 (61.9)	79.3	20.7	
	Good	945 (8.9)	81.4	18.6		374 (8.1)	84.0	16.0		571 (9.4)	79.7	20.3	
**Total**		10,676 (100.0)	8392 (78.6)	2284 (21.4)		4625 (100.0)	3736 (80.8)	889 (19.2)		6051 (100.0)	4656 (76.9)	1395 (23.1)	

^a^Age for 17 participants are missing: 6 for males and 11 for females

Referring to Table 2, the results of *χ*^*2*^ tests suggested that SSQ was associated with gender, age, religious belief, residence area, from single parent family or single child family or not, paternal or maternal education level, and household economic status (*P* < 0.05). A lower SSQ (score 1–2.9) was found in males, students aged 26 years old and above, and those having religious beliefs, living in rural areas, from single parent families, or non-single child families. The finding also showed in students whose parents’ education levels were primary or below and those from families with poorer economic statuses (P < 0.05). For males, college students who are from single child families, whose parents have higher education levels, and with better household economic statuses are more likely to have high SSQ, but college students from single parent families have lower SSQ (*P* < 0.05); For females, except above significant variables for males, college students with higher age, without religious beliefs and those from rural areas also had higher SSQ (*P* < 0.05).

**Table 2:** Demographic characteristic of participants by perceived social support quality

**Demographics**		**Total**					**Males**					**Females**				
		**Sample size (%)**	**Low**	**Moderate**	**High**	***P***	**Sample size (%)**	**Low**	**Moderate**	**High**	***P***	**Sample size (%)**	**Low**	**Moderate**	**High**	***P***
**Age (years old)**^a^	< 18	680 (6.4)	1.8	33.5	64.7	< 0.001	271 (5.9)	2.6	33.6	63.8	0.116	409 (6.8)	1.2	33.5	65.3	0.006
	18–21	8136 (76.3)	1.7	35.7	62.7		3650 (78.9)	2.1	38.2	59.6		4486 (74.1)	1.3	33.6	65.1	
	22–25	1640 (15.4)	1.6	30.5	67.9		612 (13.2)	1.8	36.3	61.9		1028 (17.0)	1.5	27.1	71.4	
	≥ 26	203 (1.9)	2.5	25.6	71.9		86 (1.9)	3.5	24.4	72.1		117 (1.9)	1.7	26.5	71.8	0.411
**Ethnicity**	Han	9476 (88.8)	1.7	34.6	63.7	0.985	4205 (90.9)	2.2	37.1	60.7	0.259	5217 (87.1)	1.3	32.5	66.2	
	Minority	1200 (11.2)	1.8	34.7	63.6		420 (9.1)	1.7	41.0	57.4		780 (12.9)	1.8	31.3	66.9	
**Religious belief**	No	10,097 (94.6)	1.6	34.3	64.1	0.001	4345 (93.9)	2.2	37.1	60.7	0.155	5752 (95.1)	1.2	32.1	66.6	< 0.001
	Yes	579 (5.4)	2.9	39.7	57.3		280 (6.1)	2.1	42.9	55.0		299 (4.9)	3.7	36.8	59.5	
**Residence area**	Rural	4978 (46.6)	1.9	38.0	60.1	< 0.001	2326 (50.3)	2.1	35.8	62.0	0.061	3372 (55.7)	1.1	28.7	70.2	< 0.001
	Urban	5698 (53.4)	1.5	31.6	66.8		2299 (49.7)	2.2	39.1	58.7		2679 (44.3)	1.6	37.0	61.4	
**From a single parent family or not**	Yes	817 (7.7)	3.2	41.5	55.3	< 0.001	317 (6.9)	3.8	41.6	54.6	0.022	500 (8.3)	2.8	42.4	55.8	< 0.001
	No	9859 (92.3)	1.6	34.0	64.4		4308 (93.1)	2.0	37.2	60.8		5551 (91.7)	1.2	31.6	67.2	
**From a single child family or not**	Yes	4931 (46.2)	1.7	31.6	66.7	< 0.001	2397 (51.8)	2.2	34.0	63.8	< 0.001	2534 (41.9)	1.2	29.4	69.4	< 0.001
	No	5745 (53.8)	1.7	37.1	61.2		2228 (48.2)	2.2	41.2	56.7		3517 (58.1)	1.4	34.5	64.0	
**Paternal education level**	Elementary school and below	1558 (14.6)	2.2	40.0	57.8	< 0.001	711 (15.4)	2.8	42.1	55.1	0.002	847 (14.0)	1.7	38.3	60.1	< 0.001
	Junior high school	3872 (36.3)	1.6	37.2	61.2		1745 (37.7)	2.0	38.9	59.1		2127 (35.2)	1.3	35.9	62.8	
	High/Secondary school	2739 (25.7)	1.6	32.9	65.4		1152 (24.9)	2.2	36.7	61.1		1587 (26.2)	1.3	30.2	68.6	
	College and above	2507 (23.5)	1.6	29.0	69.5		1017 (22.0)	2.0	32.7	65.3		1490 (24.6)	1.3	26.4	72.3	
**Maternal education level**	Elementary school and below	2742 (25.7)	2.0	39.6	58.4	< 0.001	1275 (27.6)	2.4	39.4	58.3	< 0.001	1467 (24.2)	1.6	39.9	58.5	< 0.001
	Junior high school	3711 (34.8)	1.5	36.0	62.5		1646 (35.6)	1.9	38.5	59.7		2065 (34.1)	1.2	34.0	64.8	
	High/Secondary school	2395 (22.4)	1.5	32.2	66.3		983 (21.3)	2.4	39.3	58.3		1412 (23.3)	0.9	27.2	71.9	
	College and above	1828 (17.1)	1.9	27.4	70.8		721 (15.6)	2.1	29.4	68.5		1107 (18.3)	1.7	26.0	72.3	
**Household economic status**	Poor	3358 (31.5)	2.5	41.6	55.9	< 0.001	1624 (35.1)	3.0	42.6	54.4	< 0.001	1734 (28.7)	2.0	40.7	57.4	< 0.001
	General	6373 (59.7)	1.3	32.9	65.8		2627 (56.8)	1.6	36.0	62.4		3746 (61.9)	1.0	30.8	68.2	
	Good	945 (8.9)	1.7	21.0	77.4		374 (8.1)	2.1	25.7	72.2		571 (9.4)	1.4	17.9	80.7	
**Total**		10,676 (100.0)	181 (1.7)	3692 (34.6)	6803 (63.7)		4625 (100.0)	100 (2.2)	1733 (37.5)	2792 (60.4)		6051 (100.0)	81 (1.3)	1959 (32.4)	4011 (66.3)	

^a^Age for 17 participants are missing: 6 for males and 11 for females

### Influencing factors of mental health

As shown in Tables 3, regression analysis indicated that aged 18–21 years old, having religious belief, from single parent families, maternal education level is college and above, and drinking alcohol were associated with poorer mental health statuses (odds ratios (ORs) were between 1.191 and 1.291, all *P* < 0.05). On the contrary, the male college students, and those who having general and higher household economic status, regular work-rest routine, sleep duration ≥ 7 h, moderate and high SSQ were more likely to have better mental health statuses (ORs ranged from 0.251 to 0.766, all *P* < 0.05). Among them, SSQ was one of the most significant influencing factors for students’ mental health problems (moderate *vs.* low: OR = 0.528, 95% CI = 0.387–0.720; high *vs.* low: OR = 0.251, 95% CI = 0.184–0.342).

**Table 3:** Multivariate logistic regression analyses for the influencing factors of mental health among ALL college students (*N* = 10,676)

**Variables**		***OR***	**95% *CI***		***P***
			**LL**	**UL**	
**Gender (ref. = Female)**	Male	0.760	0.684	0.844	< 0.001
**Age (years old) (ref. = < 18)**	18–21	1.291	1.047	1.592	0.017
	22–25	1.253	0.987	1.591	0.063
	≥ 26	1.008	0.649	1.566	0.971
**Religious belief (ref. = No)**	Yes	1.234	1.008	1.510	0.042
**From a single parent family or not (ref. = No)**	Yes	1.191	1.004	1.413	0.045
**Maternal education level (ref. = Elementary school and below)**	Junior high school	0.913	0.801	1.042	0.176
	High/Secondary school	1.001	0.845	1.185	0.991
	College and above	1.259	1.018	1.558	0.033
**Household economic status (ref. = Lower)**	General	0.705	0.625	0.790	< 0.001
	Higher	0.679	0.550	0.839	< 0.001
**Work-rest routine (ref. = Never/Seldom)**	Sometimes	0.766	0.674	0.872	< 0.001
	Usually/Always	0.543	0.480	0.613	< 0.001
**Sleep duration (hours) (ref. = < 7)**	7–9	0.697	0.627	0.774	< 0.001
	≥ 9	0.708	0.550	0.911	0.007
**Alcohol drinking (ref. = No)**	Yes	1.239	1.083	1.416	0.002
**Social support quality (ref. = Low level)**	Moderate level	0.528	0.387	0.720	< 0.001
	High level	0.251	0.184	0.342	< 0.001

*OR* Odds ratio, *CI* Confidence interval, *LL* Low limit, *UL* Upper limit, *ref.* Reference

**Table 4:** Multivariate logistic regression analyses for the influencing factors of mental health among MALE college students (*N* = 4625)

**Variables**		***OR***	**95% *CI***		***P***
			**LL**	**UL**	
**Ethnicity (ref. = Han)**	Minority	1.292	1.006	1.660	0.045
**From a single child family or not (ref. = No)**	Yes	0.830	0.697	0.988	0.036
**Household economic status (ref. = Lower)**	General	0.737	0.619	0.878	0.001
	Higher	0.675	0.478	0.952	0.025
**Work-rest routine (ref. = Never/Seldom)**	Sometimes	0.754	0.615	0.925	0.007
	Usually/Always	0.493	0.407	0.598	< 0.001
**Sleep duration (hours) (ref. = < 7)**	7–9	0.694	0.585	0.823	< 0.001
	≥ 9	0.631	0.434	0.917	0.016
**Social support quality (ref. = Low level)**	Moderate level	0.486	0.318	0.741	0.001
	High level	0.236	0.155	0.361	< 0.001

*OR* Odds ratio, *CI* Confidence interval, *LL* Low limit, *UL* Upper limit, *ref.* Reference

**Table 5:** Multivariate logistic regression analyses for the influencing factors of mental health among FEMALE college students (*N* = 6051)

**Variables**		***OR***	**95% *CI***		***P***
			**LL**	**UL**	
**Age (years old) (ref. = < 18)**	18–22	1.460	1.114	1.915	0.006
	23–26	1.403	1.034	1.905	0.030
	≥ 26	1.184	0.673	2.083	0.558
**Religious belief (ref. = No)**	Yes	1.327	1.012	1.741	0.041
**Maternal education level (ref. = Elementary school and below)**	Junior high school	0.975	0.821	1.159	0.777
	High/Secondary school	1.040	0.833	1.298	0.728
	College and above	1.472	1.120	1.934	0.006
**Household economic status (ref. = Lower)**	General	0.684	0.588	0.795	< 0.001
	Higher	0.678	0.518	0.888	0.005
**Work-rest routine (ref. = Never/Seldom)**	Sometimes	0.781	0.661	0.922	0.004
	Usually/Always	0.578	0.492	0.678	< 0.001
**Sleep duration (hours) (ref. = < 7)**	7–9	0.697	0.610	0.797	< 0.001
	≥ 9	0.776	0.550	1.095	0.149
**Alcohol drinking (ref. = No)**	Yes	1.493	1.212	1.838	< 0.001
**Social support quality (ref. = Low level)**	Moderate level	0.596	0.376	0.947	0.028
	High level	0.280	0.177	0.443	< 0.001

*OR* Odds ratio, *CI* Confidence interval, *LL* Low limit, *UL* Upper limit, *ref.* Reference

There are gender differences in influencing factors for mental health statuses. For males, ethnic minority (OR = 1.292) was a negative influencing factors for mental health (all *P* < 0.05), while from a single child family, general and higher household economic status, work-rest routine, sleeping ≥ 7 h, moderate and high SSQ could be positive influencing factors for mental health (ORs ranged from 0.236 to 0.830, all *P* < 0.05) (Table 4). For females, 18–25 years old, having religious belief, maternal education level is college and above, and drinking alcohol were negative influencing factors for mental health (ORs ranged from 1.327 to 1.493, all *P* < 0.05), while general and higher household economic status, work-rest routine, sleeping ≥ 7 h, moderate and high SSQ could be positive influencing factors for mental health (ORs ranged from 0.280 to 0.781, all *P* < 0.05) (Table 5).

### SSQ and mental health

In Table 6, the correlation matrix suggested that SSQ was negatively associated with mental health problems (*r* = -0.182). Among the three social support sources, SSQ from family provided the strongest effect on mental health problems (*r* = -0.182), then followed by that from friends (*r* = -0.167) and that from significant others (*r* = -0.157). Within the MSPSS, the family, friends and significant others subscales were highly correlated with each other (*r* between 0.586 and 0.717) and with the overall scale (*r* between 0.780 and 0.836). Furthermore, we have analyzed the correlation between MSPSS and SSQ for both male and female college students (Tables 7 and 8).

**Table 6:** Correlation between MSPSS and GHQ-12 for ALL college students (*N* = 10,676)

**Variables**	SSQ	Subscale-family	Subscale-friends	Subscale-significant others	Mental health problems
SSQ	1.000				
Subscale-family	0.780	1.000			
Subscale-friends	0.782	0.586	1.000		
Subscale-significant others	0.836	0.656	0.717	1.000	
Mental health problem	-0.182	-0.182	-0.167	-0.157	1.000

*MSPSS* Multidimensional Scale of Perceived Social Support, *GHQ* General health questionnaire, *SSQ* Social support quality

All correlation coefficients were statistically significant at the 0.01 level

**Table 7:** Correlation between MSPSS and GHQ-12 for MALE college students (*N* = 4625)

**Variables**	SSQ	Subscale-family	Subscale-friends	Subscale-significant others	Mental health problems
SSQ	1.000				
Subscale-family	0.799	1.000			
Subscale-friends	0.800	0.617	1.000		
Subscale-significant others	0.837	0.679	0.722	1.000	
Mental health problem	-0.182	-0.175	-0.161	-0.168	1.000

*MSPSS* Multidimensional Scale of Perceived Social Support, *GHQ* General health questionnaire, *SSQ* Social support quality

All correlation coefficients were statistically significant at the 0.01 level

**Table 8:** Correlation between MSPSS and GHQ-12 for FEMALE college students (*N* = 6051)

**Variables**	SSQ	Subscale-family	Subscale-friends	Subscale-significant others	Mental health problems
SSQ	1.000				
Subscale-family	0.766	1.000			
Subscale-friends	0.766	0.560	1.000		
Subscale-significant others	0.836	0.639	0.712	1.000	
Mental health problem	-0.189	-0.190	-0.176	-0.160	1.000

*MSPSS* Multidimensional Scale of Perceived Social Support, *GHQ* General health questionnaire, *SSQ* Social support quality

All correlation coefficients were statistically significant at the 0.01 level

As presented in Table 9,  compared with low SSQ, both high and moderate SSQ could reduce the risk of mental health problems (ORs ranged from 0.183 to 0.528, *P* < 0.05). In different models of each subscale, there were significant differences in effect of different SSQs on mental health (*P*_difference_ all < 0.05). Additionally, in all three models with different adjustments, there were significant positive trends in associations between both full scale and subscales of SSQ and mental health problems (*P*_trend_ all < 0.001). Especially, in model 3, with the full adjustment, both higher and moderate SSQ had greater negative impacts on mental health problems than the low SSQ (OR = 0.251, 95% CI = 0.184–0.342; OR = 0.528, 95% CI = 0.387–0.720, respectively). In subscales, family supports had the strongest preventive effect on students’ mental health problems (high/moderate SSQ = 0.406/0.214), then followed by friends supports (0.428/0.230) and significant others supports (0.514/0.277).

**Table 9:** Multivariate logistic regression analyses for the association between social support quality and mental health statuses for ALL college students (*N* = 10,676)

**Variables**		**Unadjusted**				**Model 1**^a^				**Model 2**^b^				**Model 3**^c^			
		***OR***	**95% *CI***		***P***_**trend**_	***OR***	**95% *CI***		***P***_**trend**_	***OR***	**95% *CI***		***P***_**trend**_	***OR***	**95% *CI***		***P***_**trend**_
			**LL**	**UL**			**LL**	**UL**			**LL**	**UL**			**LL**	**UL**	
**SSQ (ref. = Low)**	Moderate	0.498	0.369	0.673	< 0.001	0.486	0.358	0.659	< 0.001	0.509	0.374	0.692	< 0.001	0.528	0.387	0.720	< 0.001
	High	0.220	0.163	0.296		0.211	0.155	0.285		0.227	0.167	0.308		0.251	0.184	0.342	
***P***_**difference**_			< 0.05				< 0.05				< 0.05				< 0.05		
**SSQ-Family (ref. = Low)**	Moderate	0.385	0.303	0.489	< 0.001	0.381	0.299	0.484	< 0.001	0.402	0.315	0.512	< 0.001	0.406	0.317	0.519	< 0.001
	High	0.187	0.147	0.237		0.183	0.144	0.233		0.198	0.156	0.253		0.214	0.167	0.274	
***P***_**difference**_			< 0.05				< 0.05				< 0.05				< 0.05		
**SSQ-Friends (ref. = Low)**	Moderate	0.400	0.308	0.521	< 0.001	0.396	0.303	0.516	< 0.001	0.417	0.319	0.545	< 0.001	0.428	0.327	0.562	< 0.001
	High	0.202	0.156	0.263		0.197	0.151	0.257		0.212	0.162	0.277		0.230	0.175	0.301	
***P***_**difference**_			< 0.05				< 0.05				< 0.05				< 0.05		
**SSQ-Significant others (ref. = Low)**	Moderate	0.504	0.384	0.662	< 0.001	0.483	0.367	0.638	< 0.001	0.502	0.379	0.663	< 0.001	0.514	0.388	0.682	< 0.001
	High	0.256	0.195	0.335		0.237	0.180	0.313		0.253	0.191	0.335		0.277	0.209	0.367	
***P***_**difference**_			< 0.05				< 0.05				< 0.05				< 0.05		

*OR *Odds ratio*, CI *Confidence interval*, LL* Low limit, *UL* Upper limit, *SSQ* Social support quality, *re*f. Reference

^a^Adjusted for age and gender

^b^Furthermore adjusted for ethnicity, religious belief, residence area, from a single parent family or not, from a single child family or not, household economic status, paternal and maternal education levels

^c^Furthermore adjusted for lifestyles (exercise, work-rest routine, sleep duration, smoking and alcohol drinking)

For both males and females, the positive trends in associations between both full scale and subscales of SSQ and mental health problems all remained (Tables 10 and 11). For male students, compared with low SSQ, both high and moderate SSQ reduced the risk of mental health problems (ORs ranged from 0.182 to 0.486, *P* < 0.05). For female students, both the high and moderate SSQ and SSQ from family or friends could reduce the risk of mental health problems (ORs ranged from 0.186 to 0.596, *P* < 0.05). However, the correlation between moderate SSQ from significant others and mental health problems was insignificant in all models (*P* > 0.05), but the high level SSQ from significant others was still a positive factor for mental health problems for female students (*P* < 0.05).

**Table 10:** Multivariate logistic regression analyses for the association between social support quality and mental health statuses for MALE college students (*N* = 4625)

**Variables**		**Unadjusted**				**Model 1**^a^				**Model 2**^b^				**Model 3**^c^			
		***OR***	**95% *CI***		***P***_**trend**_	***OR***	**95% *CI***		***P***_**trend**_	***OR***	**95% *CI***		***P***_**trend**_	***OR***	**95% *CI***		***P***_**trend**_
			**LL**	**UL**			**LL**	**UL**			**LL**	**UL**			**LL**	**UL**	
**SSQ (ref. = Low)**	Moderate	0.440	0.293	0.662	< 0.001	0.447	0.297	0.675	< 0.001	0.453	0.299	0.685	< 0.001	0.486	0.318	0.741	< 0.001
	High	0.195	0.130	0.294		0.199	0.132	0.300		0.208	0.137	0.315		0.236	0.155	0.361	
***P***_**difference**_			< 0.05				< 0.05				< 0.05				< 0.05		
**SSQ-Family (ref. = Low)**	Moderate	0.368	0.257	0.526	< 0.001	0.365	0.255	0.521	< 0.001	0.372	0.260	0.534	< 0.001	0.386	0.267	0.556	< 0.001
	High	0.183	0.128	0.261		0.182	0.127	0.260		0.191	0.133	0.274		0.214	0.146	0.306	
***P***_**difference**_			< 0.05				< 0.05				< 0.05				< 0.05		
**SSQ-Friends (ref. = Low)**	Moderate	0.350	0.242	0.507	< 0.001	0.355	0.245	0.515	< 0.001	0.372	0.256	0.543	< 0.001	0.388	0.265	0.569	< 0.001
	High	0.185	0.128	0.268		0.188	0.129	0.273		0.201	0.137	0.293		0.222	0.151	0.326	
***P***_**difference**_			< 0.05				< 0.05				< 0.05				< 0.05		
**SSQ-Significant others (ref. = Low)**	Moderate	0.377	0.265	0.537	< 0.001	0.376	0.263	0.538	< 0.001	0.381	0.266	0.546	< 0.001	0.403	0.279	0.581	< 0.001
	High	0.192	0.134	0.274		0.192	0.134	0.274		0.199	0.139	0.287		0.223	0.154	0.323	
***P***_**difference**_			< 0.05				< 0.05				< 0.05				< 0.05		

*OR *Odds ratio*, CI *Confidence interval*, LL* Low limit, *UL* Upper limit, *SSQ* Social support quality, *ref.* reference

^a^Adjusted for age and gender

^b^Furthermore adjusted for ethnicity, religious belief, residence area, from a single parent family or not, from a signal child family or not, household economic status, paternal and maternal education levels

^c^Furthermore adjusted for lifestyles (exercise, work-rest routine, sleep duration, smoking and alcohol drinking)

**Table 11:** Multivariate logistic regression analyses for the association between social support quality and mental health statuses for FEMALE college students (*N* = 6051)

**Variables**		**Unadjusted**				**Model 1**^a^				**Model 2**^b^				**Model 3**^c^			
		***OR***	**95% *CI***		***P***_**trend**_	***OR***	**95% *CI***		***P***_**trend**_	***OR***	**95% *CI***		***P***_**trend**_	***OR***	**95% *CI***		***P***_**trend**_
			**LL**	**UL**			**LL**	**UL**			**LL**	**UL**			**LL**	**UL**	
**SSQ (ref. = Low)**	Moderate	0.540	0.346	0.843	< 0.001	0.535	0.340	0.841	< 0.001	0.582	0.369	0.918	< 0.001	0.596	0.376	0.947	< 0.001
	High	0.228	0.147	0.356		0.228	0.145	0.357		0.254	0.162	0.401		0.280	0.177	0.443	
***P***_**difference**_			< 0.05				< 0.05				< 0.05				< 0.05		
**SSQ-Family (ref. = Low)**	Moderate	0.397	0.287	0.548	< 0.001	0.395	0.285	0.546	< 0.001	0.427	0.307	0.593	< 0.001	0.433	0.310	0.604	< 0.001
	High	0.187	0.135	0.257		0.186	0.134	0.256		0.206	0.148	0.286		0.224	0.161	0.313	
***P***_**difference**_			< 0.05				< 0.05				< 0.05				< 0.05		
**SSQ-Friends (ref. = Low)**	Moderate	0.440	0.303	0.639	< 0.001	0.435	0.298	0.634	< 0.001	0.458	0.313	0.671	< 0.001	0.465	0.316	0.685	< 0.001
	High	0.209	0.144	0.304		0.209	0.144	0.305		0.226	0.154	0.330		0.242	0.165	0.356	
***P***_**difference**_			< 0.05				< 0.05				< 0.05				< 0.05		
**SSQ-Significant others (ref. = Low)**	Moderate	0.692	0.449	1.068	< 0.001	0.693	0.447	1.076	< 0.001	0.745	0.478	1.161	< 0.001	0.759	0.484	1.190	< 0.001
	High	0.328	0.213	0.505		0.331	0.214	0.512		0.368	0.237	0.573		0.402	0.257	0.629	
***P***_**difference**_			< 0.05				< 0.05				< 0.05				< 0.05		

*OR *Odds ratio*, CI *Confidence interval*, LL* Low limit, *UL* Upper limit, *SSQ* Social support quality, *ref.* reference

^a^Adjusted for age and gender

^b^Furthermore adjusted for ethnicity, religious belief, residence area, from a single parent family or not, from a signal child family or not, household economic status, paternal and maternal education levels

^c^Furthermore adjusted for lifestyles (exercise, work-rest routine, sleep duration, smoking and alcohol drinking)

## Discussion

A total of 10,676 college students participated in this study and the prevalence of mental health problems was 21.4%. Although this result was lower than the finding (28.0%) from a survey conducted among Finnish university students [32], it was higher than results of a cross-nation mental health survey (20.3%) [8] and another one (19.0%) from Hungary [33], all above studies were conducted among college students using the GHQ-12. These differences might be attributed to racial, cultural, and socio-demographic disparities [34]. With the increase of SSQ, the risk of mental health problems among college students showed a significant decreasing trend, suggesting that improving the SSQ could be an effective and practical method to prevent mental health problems of college students.

Compared with liberal education in western developed countries, China follows a relatively conservative education model. Under this model, schools are prone to pursue attractive academic achievements rather than students’ quality-oriented education and healthy psychological development [35]. Heavy academic burden and insufficient healthcare on mental health lead to negative mental health statuses among Chinese students. However, because mental health problems could be long-lasting throughout the entire education period, plus the transitional period of psychological development, which is sensitive to surrounding environment, lifestyles and social supports, the mental health problems are prominent among college students.

Mental health problems are associated with various factors. Generally, the female are better at recognizing emotions and expressing themselves more easily, along with higher rates of treatment engagement and mental health symptomatology reporting [22, 36]. However, privacy/stigma concerns were more prominent for males [37]. Thus, both male and female college students’ mental health problems should attach enough attention. Compared with the older college students, the freshmen faced confused lifetime planning, cash-strapped living, and less social experience, which made them anxious and stressful. We found that minority college students seemed to have higher risk of having mental health problems than Han college students, which is similar to previous evidences [38]. This finding could be explained by “culture shock”. The minority college students not only be away from home and enter a new environment, but also face the impact of different cultural factors such as language, diet, lifestyle, and values, which might arouse their acculturation stresses and cause mental health problems ultimately [39].

Mother plays a pivotal role in the development of children’s mental health [40, 41]. Generally, mothers holding college degrees or above will be employed, and the work will take up a lot of their time and energy. Combining with the fact that fathers are also busy working, who makes up the majority of labor market in China, their children might be more vulnerable to mental health problems because their parents have less time and energy to accompany and care for them. Alcohol drinking was negatively associated with mental health. Students who drank alcohol tended to have decreased sensitivity, intense emotions, and interpersonal conflicts, which eventually increased the risk of mental health problems [42]. However, the effect between alcohol drinking and mental health problems could be bi-directional, college students opts to cope with their depression, stress and other mental health problems by drinking, which warrants further studies.

Better household economic status could protect students from having mental health problems, which aligned with previous evidences [43]. Coupled with the increasing self-esteem of college students, the gap between subjective demands and objective facts of poor economic condition contributed to psychological imbalance and ultimately induced mental health problems [44]. Students following regular work-rest schedule had significant lower risk of mental health problems. This finding was partly supported by a study based on the UK Bio-bank, where circadian disruption was reliably associated with various adverse mental health outcomes [45]. However, the age range of its study sample is 37–73 years old, the result might be not applicable for college students, and further studies are needed to confirm such relationship. In addition, sleeping seven hours and longer would decline the risk of mental health problems significantly, which was consistent with previous evidences [46, 47]. Adequate sleep could preserve the homeostasis of affective brain, and optimally prepare next-day emotional functioning, leading to a stable and healthy mental status [48, 49].

Adequate high-quality social supports could give individuals comfortable mental consolation and a sense of security, which benefits college students to keep a healthy psychological status [50]. Due to the conservative family values and collectivist nature of Chinese society, family ties were deemed as the most important social relationships [51]. People suffering or experiencing mental health problems often create the feelings of stigma or shame. In this case, the family could play a key role in guiding family members with psychological problems to receive treatment, and making them healthy through active interventions [52]. However, several studies listed friends as the most important source of social supports, ahead of family and significant others [53]. The reason might be that most college students lived with friends in the campus rather than their family members, friends could discover each other’s psychological changes timely, and social supports from friends could offer sufficient mental assistance. However, in this study, SSQ from family, friends, or significant others all could improve college students’ mental health statuses to some extent. These findings informed that university administrators and teachers should improve students’ SSQ, such as regular psychological counseling, advocating harmonious relationships, especially encouraging students to keep in touch with their families and friends for high-quality social supports, to prevent mental health problems in college students better.

### Strengths and limitations

There were several strengths in this study. Firstly, we included 10,676 students in this study via the multi-stage random cluster sampling. It was a big sample size among relevant studies, guaranteeing the results were credible to some extent. Secondly, we obtained some important findings. For example, there was a decreasing trend of having mental health problems with the improvement of SSQ, which further supplemented and confirmed the influencing factor network of college students’ mental health, and provided statistical data for international comparisons on this topic.

However, some limitations should be acknowledged. Firstly, this study was a cross-sectional design, the results only suggested the observational correlation rather than the causality between SSQ and mental health problems. Secondly, students’ SSQ and mental health statuses were collected through self-reported, which might be a potential source of information bias. Thirdly, some classifications on socio-demographic and lifestyle variables were simple, which, to some extent, limited further analyses on the impact of specific socio-demographic characteristics or lifestyles on individual mental health status. Fourthly, previous investigators have identified differences in mental health literacy between students who were enrolled in different majors [54]. However, we did not considering the effect of the discipline settings or specialty background on mental health in college students, which might be another mixed factor. Relevant studies in this topic could be conducted in the future. Finally, this study was conducted in Wuhan city, which might limit the generality to other regions. However, college students in Wuhan were from across the country, which could make up for the lack of sample representation to some extent.

## Conclusion

Besides socio-economic and lifestyle factors, social support is a positive and critical factor for mental health of college students. The higher SSQ, especially that from the family, could be better in preventing mental health problems than those from friends or significant others. These findings could provide valuable and practical clues for the prevention of mental health problems among college students.

## Supplementary Information

**Additional file 1.**

## Data Availability

All data generated or analyzed during this study are included in this manuscript. However, the datasets generated and/or analyzed in present study are not publicly available due the data is collected by the project team, we can use it with authorization, but cannot share it publicly, but are available from the corresponding author on reasonable request.

## Abbreviations

SSQ: Social support quality
MSPSS: Multidimensional Scale of Perceived Social Support
GHQ-12: The 12-items General Health Questionnaire
SD: Standard deviation
OR: Odds ratio
Ref: Reference
