# Supplementary material for: Socio-demographic characteristics, lifestyles, social support quality and mental health in college students: a cross-sectional study
Source: BMC Public Health. 2022 Aug 20;22:1583. doi: 10.1186/s12889-022-14002-1 (PMC9392273; doi:10.1186/s12889-022-14002-1)
Supplement: Supplementary file 1 — Additional file 1. [file 12889_2022_14002_MOESM1_ESM.docx]

**Investigation for Health Service Demand and Utilization of University/College Students**

Dear Students:

In order to understand the physical and mental health of university/college students, optimize the health service model in school hospital, and provide health services for college students better, we carry out this investigation. The investigation is anonymous and will not reveal your personal privacy. Please complete the questionnaire carefully based on your actual views and experiences. The information of your answers will be kept confidential. There is no right or wrong answer to any question. Thank you for your cooperation!

(Please tick "√" on the serial number of the corresponding answer)

**Basic situation**

1. Gender: 1) Male 2) Female

2. Age: ______ years old

3. Height: ______ cm

4. Weight: ______ kg

5. Nationality: 1) Han 2) Other _____________ (Please specify)

6. Your religious beliefs: 1) No 2) Buddhism 3) Catholic 4) Christianity 5) Islam

6) Other ______________ (Please specify)

6.1 If you have religious beliefs, what is your devotion to your beliefs?

1) Very high 2) Relatively high 3) Fair 4) Relatively low 5) Very low

7. What is your major:

1) Philosophy 2) Economics 3) Law 4) Education 5) Literature

6) History 7) Science 8) Engineering 9) Agriculture 10) Medicine

11) Military 12) Management 13) Art

8. The grade you are currently enrolled in is:

1) Grade 1 2) Grade 2 3) Grade 3 4) Grade 4

5) Grade 5 6) Master's degree 7) Doctoral degree

9. Type of current residence of the family:

1) Large cities (Provincial capitals, Municipalities directly under the Central Government, or special economic zones)

2) Small and medium cities (prefecture and county level)

3) Townships

4) Rural areas

10. Are you a single parent family?

1) yes 2) no

11. Are you an only child? 1) Yes 2) No, ranking at home ______ (Please specify)

12. Your father's education level:

1) Elementary school and below 2) Junior high school

3) High school / technical secondary school 4) University / junior college

5) Graduate student and above

13. Your mother's education level:

1) Elementary school and below 2) Junior high school

3) High school / technical secondary school 4) University / junior college

5) Graduate student and above

14. How do you think your family's financial situation is?

1) Very good 2) Relatively good 3) Average 4) Relatively poor 5) Very poor

15. Your monthly living expenses are approximately:

1) Below 500 yuan 2) 500-999 yuan 3) 1000-1499 yuan

4) 1500-1999 yuan 5) 2000-2499 yuan 6) more than 2500 yuan

16. The sources of your living expenses. Please sort it by importance _____________:

1) Parents 2) Relatives and friends 3) Part-time work

4) Student Loans 5) Scholarships 6) Others _____________ (Please specify)

**Healthy living behavior**

17. Can you do vigorous exercise for about 30 minutes each time (excluding warm-up time), at least 3 times a week?

1) Never 2) Occasionally 3) About half of the time 4) Often 5) All the time

18. Do you have 30-60 minutes of chronic aerobic exercise (e.g. jogging, walking) 3 times a week?

1) Never 2) Occasionally 3) About half of the time 4) Often 5) All the time

19. Do you eat breakfast every day?

1) Never 2) Occasionally 3) About half of the time 4) Often 5) All the time

20. Do you drink at least 800ml of water every day?

1) Never 2) Occasionally 3) About half of the time 4) Often 5) All the time

21. Do you work and rest regularly?

1) Never 2) Occasionally 3) About half of the time 4) Often 5) All the time

22. In past 3 months, you have slept ______hours and ______minutes **per day**.

23. In past 3 months, your smoking status:

1) At least 1 cigarette per day 2) At least 1 cigarette per week

3) Smoking less than 1 cigarette per week 4) No smoking behavior

24. Alcohol consumption:

24.1 How often you drink:

1) ≥ 1 per week 2) 1~3 times per month

3) < 1 per month 4) No alcohol (Please skip to 25)

24.2 How much do you usually drink each time: (take one can of beer or one glass of wine or 500 kg liquor as a unit)

1) One unit or less 2) 2~4 units 3) ≥5 units

25. Do you think keeping physical or mental health is important for college students / graduates?

1) Very important 2) Somewhat 3) Generally important

4) Not very important 5) Not at all

26. Your attention to knowledge related to physical and mental health:

1) Very concerned 2) Frequently 3) Occasionally 4) Rarely 5) Not at all

**Demand for health services**

27. How do you think your physical condition is?

1) Very good 2) Relatively good 3) Fair 4) Relatively poor 5) Very poor

28. Have you experienced any illness or injury in last two weeks?

1) Yes, new illness or injury 2) Yes, acute illness or injury

3) Yes, chronic illness 4) No (Please skip to 29)

28.1 Have you seen a doctor if you have been sick in last two weeks?

1) Yes 2) No (Please skip to 29)

28.2 Your preferred medical unit is:

1) Private outpatient clinic 2) Community service center 3) School hospital

4) Secondary or tertiary hospitals 5) Other ____________ (please specify)

28.3 If you are sick but do not see a doctor, what is the reason? (Multiple choice)

1) The symptom is mild 2) Buy medicine and self-medicates 3) No time

4) High medical expenses 5) Poor hospital service 6) Inconvenient

7) Personal privacy reasons 8) Other _____________ (Please specify)

29. In the last year, have you suffered from diseases requiring hospitalization? 1) yes 2) no

30. Do you suffer from chronic diseases that require long-term examination or medication for more than one year?

1) Yes (Please specify _____________) 2) No

31. If the doctor in the school hospital cannot diagnose or treat your disease, will they refer you to a higher-level hospital in time?

1) Always 2) Often 3) Occasionally 4) Never 5) Don't know

32. Are you satisfied with the referral process?

1) Never been referred 2) Very satisfied 3) Somewhat satisfied

4) Fair 5) Not very satisfied 6) Very dissatisfied

33. Can you able to meet your basic medical needs through referrals?

1) Always 2) Often 3) Sometimes 4) Often not 5) Never

34. Do you think the medical service capacity of the school hospital can meet your basic diagnosis and treatment needs?

1) Fully 2) Basically 3) Partially 4) Completely unsatisfied 5) Don't know

34.1 What are you most dissatisfied with about the school's medical security services? (up to 3 optional)

1) Low technical level 2) Poor equipment conditions 3) Few types of drugs

4) Poor service attitude 5) Unreasonable charges 6) High medical expenses

7) Cumbersome procedures for seeing a doctor 8) Long waiting time

9) Poor environmental conditions 10) Provision of unnecessary services (including medicines and inspections) 11) None 12) Other ______(Please specify)

35. Please rank the degree of your need for the following services and fill in the number in the horizontal line _____________

1) Outpatient service 2) Inpatient service 3) Health education 4) Regular physical

examination 5) Psychological consultation 6) First aid knowledge training

**Medical insurance**

36. Are you currently covered by health insurance? 1) Yes 2) No 3) Don't know

37. How well do you understand the basic medical insurance system for college students?

1) Very well 2) Somewhat 3) A little bit 4) Very little 5) Don't know

38. Do you know the medical treatment procedures for college students after enrolling in medical insurance?

1) Very well 2) Somewhat 3) A little bit 4) Very little 5) Don't know

39. Do you understand the transfer process for college students after enrolling in medical insurance?

1) Very well 2) Somewhat 3) A little bit 4) Very little 5) Don't know

40. Have you ever been reimbursed by medical insurance due to illness?

1) Yes 2) No 3) Don't know

41. Do you think enrolling in medical insurance will help you a lot?

1) Very large 2) Relatively large 3) Average 4) Relatively small 5) Very small

42. Are you satisfied with the medical insurance you enjoy now?

1) Yes (Please skip to 43) 2) No

42.1 If you are not satisfied with your current medical insurance, what are the main reasons?

1) The reimbursement ratio is too low 2) The reimbursement procedure is cumbersome

3) The convenience of medical treatment is restricted 4) Others

43. Will you be covered by health insurance while you are in school?

1) Yes (Please skip to 44) 2) No

43.1 If you select "No", what is your reason for not participating?

1) Healthy 2) Economic difficulties 3) The reimbursement procedures are troublesome

4) The reimbursement ratio is low 5) Other ____________ (Please specify)

**Mental health status (GHQ-12)**: Please choose the option that best matches your situation in the last four weeks:

44. Insomnia due to anxiety (anxiety/depression)

1) Not at all 2) Same as usual 3) A little more than usual 4) Much more than usual

45. Feeling stressed all the time (anxiety/depression)

1) Not at all 2) Same as usual 3) A little more than usual 4) Much more than usual

46. ​​Ability to concentrate when doing things (anxiety/depression)

1) Better than usual 2) Same as usual 3) Worse than usual 4) Much worse than usual

47. Feeling useful in life

1) Better than usual 2) Same as usual 3) Worse than usual 4) Much worse than usual

48. Be able to face your problems

1) Better than usual 2) Same as usual 3) Worse than usual 4) Much worse than usual

49. Feeling able to make decisions about what happens

1) Better than usual 2) Same as usual 3) Worse than usual 4) Much worse than usual

50. Feeling unable to overcome difficulties

1) Not at all 2) Same as usual 3) A little more than usual 4) Much more than usual

51. Overall good mood (anxiety/depression)

1) Better than usual 2) Same as usual 3) Worse than usual 4) Much worse than usual

52. Being able to enjoy everyday life

1) Better than usual 2) Same as usual 3) Worse than usual 4) Much worse than usual

53. Feeling unhappy or down (anxiety/depression)

1) Not at all 2) Same as usual 3) A little more than usual 4) Much more than usual

54. Lose confidence in yourself

1) Not at all 2) Same as usual 3) A little more than usual 4) Much more than usual

55. Think of yourself as a worthless person

1) Not at all 2) Same as usual 3) A little more than usual 4) Much more than usual

**Cognition and utilization of mental health services**

56. Overall, to what extent do you feel you have had psychological distress in the last month?

1) Severe 2) Major 3) Moderate 4) Mild 5) No distress at all

57. When you encounter psychological distress, your usual solution is to:

1) Exercise 2) Recreation 3) Smoking 4) Drinking 5) Eating 6) Being alone

7) Find someone to talk to and ask for help 8) Study hard 9) Others

58. Do you have any psychological troubles that you could not solve?

1) Yes 2) No (Please skip to 59)

58.1 If yes, have you received professional mental health counseling or treatment?

1) Yes 2) No (Please skip to 58.1.2)

58.1.1 Do you think psychological counseling or treatment is helpful for relieving your psychological distress?

1) Very helpful 2) Somewhat helpful 3) Fair 4) Slightly helpful 5) No helpful

58.1.2 If no, why did you not seek psychological counseling or treatment? (Multiple choice)

1) Don't know about counseling or therapy 2) Don't know how to seek

3) Think that is time-consuming 4) Think that is not helpful

5) Prefer to choose other options, such as confiding in friends or family

6) Worry that counseling or therapy will have a negative impact on my life or studies

7) I am ashamed of being in counseling or therapy

8) I am worried that people around me will look at me differently when they find out

9) Other

59. If you encounter psychological troubles that you cannot solve in the future,

59.1 If you decide to seek help from someone, you will first choose:

1) Family 2) Friends 3) Classmates 4) Teachers 5) Netizens 6) Gods

7) Counseling/Therapeutic Professionals 8) Doctors 9) Not asking help 10) Others

59.2 If you want to seek professional services, how likely do you think the availability?

1) Unlikely 2) Unlikely 3) Somewhat 4) More likely 5) Very likely

**Social support status (MSPSS)**

60. A special person (relatives, teachers, classmates) will be around when I am in need.

1) Strongly disagree 2) Strongly disagree 3) Slightly disagree 4) Neutral

5) Slightly agree 6) Strongly agree 7) Strongly agree

61. I can share joys and sorrows with a special person (family members, teachers, classmates).

1) Strongly disagree 2) Strongly disagree 3) Slightly disagree 4) Neutral

5) Slightly agree 6) Strongly agree 7) Strongly agree

62. My family can help me concretely.

1) Strongly disagree 2) Strongly disagree 3) Slightly disagree 4) Neutral

5) Slightly agree 6) Strongly agree 7) Strongly agree

63. I can get emotional help and support from my family when I need it.

1) Strongly disagree 2) Strongly disagree 3) Slightly disagree 4) Neutral

5) Slightly agree 6) Strongly agree 7) Strongly agree

64. Special persons (family, teachers, classmates) are the real source of comfort when I am in trouble

1) Strongly disagree 2) Strongly disagree 3) Slightly disagree 4) Neutral

5) Slightly agree 6) Strongly agree 7) Strongly agree

65. My friends can really help me

1) Strongly disagree 2) Strongly disagree 3) Slightly disagree 4) Neutral

5) Slightly agree 6) Strongly agree 7) Strongly agree

66. I can count on my friends in times of trouble

1) Strongly disagree 2) Strongly disagree 3) Slightly disagree 4) Neutral

5) Slightly agree 6) Strongly agree 7) Strongly agree

67. I can talk to my family about my problems

1) Strongly disagree 2) Strongly disagree 3) Slightly disagree 4) Neutral

5) Slightly agree 6) Strongly agree 7) Strongly agree

68. My friends can share joy and sorrow with me

1) Strongly disagree 2) Strongly disagree 3) Slightly disagree 4) Neutral

5) Slightly agree 6) Strongly agree 7) Strongly agree

69. There are a special person in my life (family, teachers, classmates) who care about my feelings

1) Strongly disagree 2) Strongly disagree 3) Slightly disagree 4) Neutral

5) Slightly agree 6) Strongly agree 7) Strongly agree

70. My family is willing to help me make decisions

1) Strongly disagree 2) Strongly disagree 3) Slightly disagree 4) Neutral

5) Slightly agree 6) Strongly agree 7) Strongly agree

71. I can discuss my problems with friends

1) Strongly disagree 2) Strongly disagree 3) Slightly disagree 4) Neutral

5) Slightly agree 6) Strongly agree 7) Strongly agree

**AIDS Knowledge, Belief and Practice**

72. Can AIDS be cured at present?

1) Yes 2) No 3) Don't know

73. Which of the following is the transmission route of AIDS? (Multiple choice)

1) Mosquito bites 2) Shared syringes 3) Shared razors 4) Handshakes/Hugs

5) Unprotected sex 6) Eating together 7) Mother-to-child transmission

8) Coughing 9) Transfusion of HIV-containing blood products

74. Which of the following are the ways to prevent AIDS? (Multiple choice)

1) Taking contraceptive pills 2) Washing vagina after sex 3) Using condoms correctly

4) Taking antibiotics 5) Preserving moral integrity

75. Are there any institutions in your city that can provide free HIV counseling and monitoring?

1) Yes 2) No 3) Don't know

76. Do you support premarital sex?

1) Support 2) Disagree 3) It depends on the situation/not good

77. Will you use condoms if you have sex?

1) Resolutely use it every time 2) Use it as much as possible

3) It doesn't matter 4) Use it as little as possible 5) Refuse to use

78. Do you agree with the statement that "AIDS is far away from me, none of my business"?

1) Strongly agree 2) Somewhat agree 3) Generally agree

4) Do not really agree 5) Strongly disagree

79. If your relative has AIDS, are you willing to continue to communicate with him/her?

1) Continue to communicate as before and give as much help as you can

2) Help but minimize interactions 3) Cut off all contact with him/her

80. What would you do if you had AIDS?

1) Seeking treatment and help 2) Hide the illness and live as usual 3) Give up life

4) Spreading the virus and infecting other people

81. If you think you may have a reproductive tract disease or a sexually transmitted disease, do you go to the hospital immediately?

1) Yes 2) Not necessarily 3) No

82. Which channels did you get your knowledge about AIDS? (Multiple choice)

1) TV/Radio 2) Internet 3) Books/Newspapers

4) Promotional materials/publicity columns 5) School health education

6) Counseling services 7) Doctors 8) Friends/classmates/family 9) Others

**The state of mental health education in colleges and universities**

83. Does your school offer a course on mental health education for college students?

1) Yes 2) No 3) Don't know

84. The main forms of mental health education work you received at your school were:

1) Not accepted 2) Required courses 3) Elective courses 4) Special lectures

5) Extracurricular activities 6) Group counseling 7) Individual interviews

8) Telephone consultation 9) Online consultation

10) Psychological clinic or counseling institution in school hospital 11) Other __________

85. Does your school have a special place to carry out mental health education?

1) Yes 2) No

86. Do you think mental health educations carried out are of great help to you?

1) Very large 2) Relatively large 3) Average 4) Relatively small 5) Very small

87. How do you think the necessary of the mental health education in university/college?

1) Not necessary 2) Not much necessary 3) It does not matter

4) Relatively necessary 5) Very necessary

The name of the university/college you are attending _____________
